# Parallel Architecture: From Problems and Mysteries to Solutions and Explanations

**DOI:** 10.1111/tops.70010

**Published:** 2025-05-20

**Authors:** Peter W. Culicover, Giuseppe Varaschin

**Affiliations:** ^1^ Department of Linguistics The Ohio State University; ^2^ Department of Linguistics University of Washington; ^3^ Institute for German Language and Linguistics Humboldt‐Universität zu Berlin

**Keywords:** Simpler syntax, Constructions, Ellipsis, Binding, Coreference, Complexity

## Abstract

We argue that Jackendoff's Parallel Architecture (PA) is the right way to think about the architecture of the language faculty. The critical property of this architecture is that it allows for genuine explanation by allocating different aspects of linguistic phenomena to appropriate corresponding representations and capacities. The PA forms the basis of a minimalist explanatory program for linguistic theory in the form of Simpler Syntax, emphasizing its constructional approach to syntax and the independence of semantics, phonology, and nonlinguistic cognitive systems.

## Abstract 2

We argue for the explanatory power of the Parallel Architecture (PA) in the domain of language. The PA permits the precise description of every aspect of linguistic phenomena, from the very general to the very idiosyncratic; it is minimalist, in that it requires linguistic generalizations to be expressed in terms of syntactic, semantic, phonological and discourse representations; it allows for linguistic phenomena to be properly linked to non‐linguistic cognition; and it illuminates which parts of language are universal, which are subject to variation, and the dimensions and sources of variation.

## 1 Introduction: The parallel architecture

We argue for the explanatory power of the Parallel Architecture (PA) (Jackendoff, 1997, 2002; Jackendoff & Audring, 2020, i.a.) in the domain of language. We consider phenomena that have been seen as problems and mysteries in conventional approaches to linguistic theory. We argue that formulating grammars in the PA offers novel solutions and explanations that are not available in conventional approaches.

The PA has several important benefits: (i) it permits the precise description of every aspect of linguistic phenomena, from the very general to the very idiosyncratic; (ii) it is minimalist, in that it requires linguistic generalizations to be expressed in terms of syntactic, semantic, phonological, and discourse representations as appropriate; (iii) it allows for linguistic phenomena to be properly linked to nonlinguistic cognition; and (iv) it illuminates which parts of language are universal, which are subject to variation, and the dimensions and sources of variation.^1^

## 2 Simpler syntax

The PA factors linguistic knowledge into distinct representational modules connected via nondirectional correspondences. Each aspect of linguistic representation is accounted for in terms of its own primitives and combinatorial principles. Correspondences range from the general, that is, “rules,” to the specific, essentially individual lexical items and idioms. The consequences for syntactic theory are explored in the context of Simpler Syntax (SiSx).

SiSx is developed in detail in Culicover and Jackendoff (2005), Jackendoff and Audring (2020), Varaschin (2021), Culicover (2021).^2^ Linguistic representations in SiSx have the form in (1), which represents the sentence *Sandy kissed Chris*. Subscripts indicate correspondences between phon(ology), syn(tax), and sem(antics). Seeking a minimalist theory, we assume that syn does not explicitly represent the phonological information that NP_1_ in (1) is pronounced as the string “Sandy” and that it precedes “kissed”— this phonological information is only represented in phon. The string “kissed” is linked to both V and T(ense), as indicated by coindices 2 and 3: it is a single piece of phonology that corresponds to two nodes in syn. The symbol ⊕ means “immediately precedes.”

(1)
$$\left[\begin{array}{cc} \mathrm{PHON} & {\left[{\mathrm{Sandy}}_{1}⊕{\mathrm{kissed}}_{2,3}⊕{\mathrm{Chris}}_{4}\right]}_{5}\\ \mathrm{SYN} & {{[}_{S}{\mathrm{NP}}_{1},T{\left[\mathrm{PAST}\right]}_{2},{[}_{\text{VP}}V_{3},{\mathrm{NP}}_{4}]]}_{5}\\ \mathrm{SEM} & {{\mathrm{past}}^{\prime }}_{2}{\left({{\mathrm{kiss}}^{\prime }}_{3}\left(\mathrm{AGENT:}{\mathrm{sandy}}_{1},\mathrm{PATIENT:}{\mathrm{chris}}_{4}\right)\right)}_{5} \end{array}\right]\;\;\;\;\;\;$$

A crucial assumption is that all aspects of sound, and in particular, the linear order of linguistic forms, are properties of phonological representations. The fact that order is represented only in phon via the immediate precedence relation does not mean that syntax is unrelated to linear order. In languages like English, there is a fairly rigid correspondence between order and syntactic structure; for example, strings that correspond to syntactic heads tend to precede strings that correspond to syntactic complements. These facts are stated in SiSx as correspondences between syn and phon.

The PA recognizes the distinction between the linguistic objects licensed by a grammar and the declarative constraints that license these objects (what Jackendoff and Audring (2020) call schemas). A well‐formed expression must be such that each of its parts satisfies at least one constraint in the grammar, in addition to the constructionally specified links among its different levels.

Example (2) shows the constraint for the lexical item *cow*. The correspondences between the phonological form “cow”, the syntactic category N, and the meaning are shown by the coindices.

(2)
$$\left[\begin{array}{cc} \mathrm{PHON} & {\mathrm{cow}}_{1}\\ \mathrm{SYN} & N_{1}\\ \mathrm{SEM} & \lambda y{\left[{\mathrm{cow}}^{\prime }\left(y\right)\right]}_{1} \end{array}\right]\;\;\;\;\;\;\;\;\;\;\;\;$$

Example (3) represents the more complex correspondence for the idiom *sell NP down the river*. Index 2 on NP_2_ shows the correspondence with variable phonological and semantic representations.

(3)
$$\left[\begin{array}{cc} \mathrm{PHON} & {\left[{\mathrm{sell}}_{1}⊕2⊕{\mathrm{down}}_{3}⊕{\mathrm{the}}_{4}⊕{\mathrm{river}}_{5}\right]}_{6}\\ \mathrm{SYN} & {{[}_{\text{VP}}V_{1},{\mathrm{NP}}_{2},{[}_{\text{PP}}P_{3},{[}_{\text{NP}}{\mathrm{Det}}_{4},N_{5}]]]}_{6}\\ \mathrm{SEM} & \lambda x{\left[{\mathrm{betray}}^{\prime }\left(\mathrm{AG}:x,\mathrm{TH}:2^{\prime }\right)\right]}_{6} \end{array}\right]\;\;\;\;\;\;\;$$

Some correspondences consist of variables alone, with no lexically specific material. These generally fall under the rubric of grammatical “rules” in theories that assume a rigid distinction between the grammar and the lexicon. An example is the schema in (4), which expresses a general correspondence for the structure of English head‐initial phrases (≪ means “precedes”).

(4)
$$\begin{array}{c} \mathrm{HEAD-INITIAL}\;\text{phrase}\\ \left[\begin{array}{cc} \mathrm{PHON} & {\left[1\ll 2\right]}_{3}\\ \mathrm{SYN} & {{[}_{\text{XP}}X_{1}^{0},{\mathrm{YP}}_{2}]}_{3} \end{array}\right] \end{array}\;\;\;\;\;\;\;\;\;\;\;\;$$

The PA is, therefore, suitable for expressing a continuum which goes from words with specific phonology, syntax, and semantics, to idioms with variable components, to fully general rules.^3^

## 3 Interpretation

We consider next how SiSx represents aspects of meaning in grammar. The PA assumes that meanings are mentally represented in terms of an algebraic representation we call semantic structure (sem) (Jackendoff, 1983, 1990). The basic primitives and combinatorial principles of sem form the innate basis for human concepts, inference, and enable judgments about truth and falsity of utterances relative to a knowledge base. Thus, the core aspects of sem are predicted to be stable across languages.^4^

Since utterances can convey an unbounded array of meanings, the semantic representation of sentences cannot be stored as indivisible chunks, but must have their own combinatorial properties and hierarchical structure, like syntax. However, SiSx views the primitives and principles governing sem as independent of syn. Motivation for this comes from the evidence of prelinguistic thought in early infants and dissociation disorders (Coetzee et al., 2022; Fedorenko & Varley, 2016; Mandler, 2004; Monti, Parsons, & Osherson, 2012). There are also reasons to believe that parts of the cognitive underpinnings of semantic structure predate humans and are found in nonlinguistic primates as well (Bermúdez, 2007; Gallistel, 2011; Schlenker, Chemla, & Zuberbühler, 2016; Sauerland & Alexiadou, 2020; Zuberbühler, Cheney, & Seyfarth, 1999).

The PA itself is neutral with respect to the format of semantic representations. We assume a logical system that is based on a small set of primitive semantic types (e.g., entities, truth‐values, and situations) and complex types defined as functions from elements of one type into another (Montague, 1974, i.a.). On this view, the basic combinatorial principle in sem is functional application.^5^

We hypothesize that phenomena whose explanation depends on structural asymmetries may be accounted for in terms of semantic representations without appealing directly to asymmetries in syntactic structure. This allows representations in syn to be simpler than in most approaches (Varaschin & Culicover, 2024). This is advantageous from an evolutionary standpoint: the more we can attribute to prelinguistic systems, the less domain‐specific knowledge we need to explain (Chomsky, 2005; Jackendoff, 2002, 2011). We first look at ellipsis, then binding, and coreference.

### 3.1 Ellipsis

ellipsis is a phenomenon in which fragments convey full linguistic content. For example, B's answer in (5) is understood in the context of A's question as “Sandy is eating pizza.”

(5)
  A: What is Sandy eating?
  B: Pizza.

The implicit meaning has traditionally been associated with invisible or deleted syntactic structure under identity with the antecedent. In (5), A's question would contain structure corresponding to the meaning “Sandy is eating x.” where *x* is what is questioned. B's answer would contain the same structure, where *pizza* is identified with *x*, along the lines of (6). The subscript *i* shows the identification of *what* in A and *pizza* B with *x*, the object of *eating*; strikeout indicates the invisibility/deletion of the identical material in B's answer.

(6)
  A: what_*i*_ [Sandy is eating $x_{i}$]
  B: pizza_*i*_ [Sandy is eating $x_{i}$]

Such accounts are workable in relatively simple cases where the form of the sentence is relatively constant across discourse. They also account for the fact that the form of the fragment appears to be constrained by the deleted structure. For example, case‐marking in German reflects the requirements of the deleted verb, as shown in (7) (Ross, 1969). The verb *schmeicheln* selects a dative NP, which is the only case possible on the elliptical *who*. In contrast, *loben* “praise” selects only accusative NPs.

(7)
  a. Er will  jemandem  schmeicheln, aber sie  wissen nicht,
    he wants someone.dat flatter    but  they   know  not
    *wer / *wen / wem.
    who.nom / who.acc /who.dat
    “He wants to flatter someone, but they don't know who.”
  b. Er will  jemanden   loben,  aber sie  wissen nicht,
    he wants someone.acc praise, but   they  know   not
    *wer / wen / *wem.
    who.nom / who.acc / who.acc

On the other hand, such accounts face the problem of specifying exactly what counts as “identical.” In (8), for example, the syntactic form of A's question contains *you*, but the deleted material in B's answer must contain *I*.

(8)
  A: What are you eating?
  B: Pizza (meaning “I am eating pizza,” not “You are eating pizza.”)

To sidestep such problems, Culicover and Jackendoff (2012) propose an approach to ellipsis that takes full advantage of the PA. What is shared by the fragment and the antecedent is not the form but the meaning. Specifically, the interpretation of the fragment is identical to that of the antecedent up to the material in the two expressions that is overtly distinguished. The computation of the meaning uses the phon and syn of the fragment to match it with an appropriate constituent in the antecedent. In this case, the NP *pizza* in (8) matches the NP *what*. The meaning of the fragment is then the sem of the antecedent with the meaning of *pizza* substituted for the matching constituent *what* in the antecedent.^6^

Crucially, matching the fragment with a constituent of the antecedent permits “indirect licensing” of the fragment (Culicover & Jackendoff, 2005, 2012); the fragment must satisfy the syntactic licensing conditions on the matching constituent in the antecedent (at least for some ellipsis constructions in some languages). In this way, indirect licensing accounts for case matching effects like (7) and similar cases without assuming invisible structure in the fragment.

### 3.2 Binding and coreference

Starting with Chomsky (1980), the semantic notion of variable binding came to be equated with a configuration like (9), where β is the binder, α is the bound form (a reflexive or a pronoun), and F is some syntactic feature that serves as a proxy for semantic identity, for example, an index or agreement feature.

(9) β binds α iff β and α are in the configuration [$\beta_{F}$… [ …$\alpha_{F}$…]]

The idea that all languages define binding in terms of the same syntactic primitives faces a number of challenges (cf. Levinson, 1991 and Varaschin, 2021 for an overview). For instance, a purely syntactic definition like (9) does not, in itself, rule out binding of proper nouns in contexts like (10):

(10) *John Smith_*i*_ was so devoid of moral sense that [he_*i*_ was willing [to hire John_*i*_'s brother in his lab]]

To rule out cases like (10), syntactic binding is constrained by binding principles (Chomsky, 1981; Safir, 2004, i.a.). (10) violates Principle C: an expression with its own reference (such as the proper name *John*) cannot be bound.

While such principles are good approximations regarding the structural conditions on anaphora, they are subject to numerous exceptions. For instance, binding is possible in (11), which is structurally parallel to (10) (Schlenker, 2005, 387).

(11) John Smith_*i*_ was so devoid of moral sense that [he_*i*_ forced [Peter Smith to hire John_*i*_'s brother in his lab]].

The PA calls for an account of examples like (10)/(11) that recognizes semantics and discourse as independent explanatory factors. For instance, a principle ruling out bound proper names is unnecessary under a semantic account of binding based on predicate logic, where only variables can be bound (Reinhart, 2006). Expressions like proper names neither correspond to nor include variables in their semantic representations; rather, they are logical constants that directly refer to some entity in speakers' (cognitive) model of the world (Jackendoff, 2002; Partee, 1986).

The conditions that govern the assignment of referents to semantic terms differ from those that govern the correspondence between syn and logical representations in sem. Varaschin, Culicover, and Winkler (in press), assuming the PA, argue that constraints on coreference are multifactorial and largely grammar‐external; they rely on systems of representation that are independent of syntax, though related to it through correspondences.

For instance, the fact that *John* and *he* cannot independently refer to the same entity in (10) but can in (11) is a consequence of a difference in the extent to which the referent associated with the pronoun is *accessible* at the point of the discourse where the proper name is encountered. Accessibility represents the ease with which a discourse referent is retrievable in a context.^7^ Thus, it is not discrete or static like the notions of syn or sem. It has to be defined in terms of a separate level of discourse structure that accommodates a gradient and dynamic ranking of referents, grounded in cognition.

Regarding (11), it has been observed that the presence of equally salient competing referents reduces the individual accessibility of each referent (Arnold & Griffin, 2007). This favors the use of proper names because they are more informative than pronouns, making it easier to identify a unique referent in an ambiguous context (Almor, 1999; Ariel, 2001). In (10), in contrast, repeating *John* causes processing load, because the referent is retrievable with a less informative expression (e.g., a pronoun).

## 4 Processing complexity

There are many instances in natural languages in which a sentence that appears to be locally well‐formed is not fully acceptable. For example, (12a) shows that a constituent in a complex structure can be questioned. In (12b), however, questioning in a comparable complex structure is unacceptable.

(12)
  a. A: Sandy told Chris [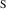 that it would be useful [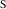 to study a complex subject]].
    B: What complex subject_*i*_ did Sandy tell Chris [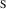 that it would be useful [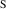 to study $t_{i}$]]?
  b. A: Sandy read [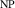 a book [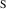 that deals with a complex subject]].
    B: *What complex subject_*i*_ did Sandy read [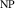 a book [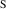 that deals with $t_{i}$]]

Another case involves freezing. Example (13a) shows that it is possible to question the complement of a preposition. But in (13b), the prepositional phrase is separated from the phrase it modifies and the comparable question is less acceptable.

(13)
  a. A: I saw [a picture [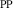 of someone interesting]] yesterday.
    B: Who did you see [a picture [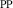 of *t*]] yesterday?
  b. A: I saw [a picture $t_{j}$] yesterday [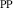 of someone interesting]_*j*_.
    B: *Who_*i*_ did you see [a picture $t_{j}$] yesterday [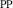 of $t_{i}$]_*j*_?

Classical syntactic theory attributes such unacceptability to the violation of island constraints on syntactic well‐formedness; extraction from islands is ungrammatical. The constraint for (12b.B) is formulated in such a way as to block extraction of a constituent α from a relative clause, that is, from the configuration [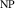 …[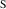 … α …]]. Similarly, the constraint for (13b.B) blocks extraction from a constituent that has itself been extracted.

At best, such constraints describe the contexts in which such unacceptability occurs but lack explanatory force. Culicover et al. (2022) propose a potentially more explanatory approach that makes crucial use of the division of labor of PA and SiSx:

**Radical Unacceptability Hypothesis (RUH)**: All judgments of reduced acceptability in cases of locally well‐formed extractions are due to processing complexity, not syntactic constraints.

On this view, syntax should only be invoked to account for phenomena when absolutely necessary, that is, when appeals to nonsyntactic linguistic representations or nonlinguistic aspects of cognition are inadequate.^8^

An early example that motivates the RUH is Miller and Chomsky's (1963) observation that the unacceptability of multiple center‐embedding is not a matter of syntactic ill‐formedness, but processing complexity. Jackendoff and Culicover (1972) applied this rationale to explain restrictions on movement out of ditransitive VPs in terms of perceptual strategies for identifying $A^{\prime }$ dependency gaps. Their basic idea was that structures like (14a) are unacceptable because the verb‐adjacent NP superficially satisfies the verb's selectional requirement, and the parser expects a gap after the preposition *to* as in (14b)—arguably a type of “garden path”; see Pritchett (1988) for a range of examples.

(14)
  a. *Who_*i*_ did Taylor give $t_{i}$ a book?
  b. Who_*i*_ did Taylor give a book to $t_{i}$?

The RUH proposes to extend this type of explanation to the extent possible. What is required in order to support the RUH is a reliable characterization of processing complexity, and confirmation that such complexity is actually at play in relevant cases. Of course, what constitutes complexity is far from straightforward. Phenomena do not come labeled with degrees, types, or sources of complexity, precise measures do not yet exist, and the precise details of the computation of sound/meaning correspondences have yet to be satisfactorily spelled out. The proposed strategy is to go from cases that are clearly syntactically well‐formed but are nevertheless not fully acceptable to some preliminary hypotheses about what might be the source of the judgment. From this, we can begin to extend and refine our hypotheses about possible contributors to complexity.

Culicover et al. (2022) suggest a number of cases where the approach appears to be promising: island constraints, freezing, and garden paths as noted above, and others, such as the following:

- Overlapping extractions: Extraction from a shifted “heavy” NP is unacceptable.^9^
  (15)
    a. You put [a picture of FDR]_*j*_ on the table.
    b. You put $t_{j}$ on the table [a picture of FDR]_*j*_.
    c. *Who_*i*_ did you put $t_{j}$ on the table [a picture of $t_{i}$]_*j*_?
- Zero‐relatives: Because there is nothing to mark the beginning of the relative clause in (16), and no initial subject, the parser cannot reliably predict the relative clause.
  (16) *He is a man under no circumstances would I give any money to *t*. (Cf. He is a man who _*i*_ under no circumstances would I give any money to $t_{i}$.)
- Weak crossover: Extracting a wh‐phrase over a pronoun that it binds can produce unacceptability (17a). But extraction of a more specific wh‐phrase improves acceptability (17b‐c).
  (17)
    a. *Who_*i*_ did his_*i*_ dean publicly denounce $t_{i}$?
    b. ??Which professor_*i*_ did his_*i*_ dean publicly denounce $t_{i}$?
    c. ?[Which distinguished molecular biologist that I used to work with]_*i*_ did his_*i*_ dean publicly denounce $t_{i}$?
    (Levine & Hukari, 2006)
  The crucial factor here is whether the discourse representation corresponding to the wh‐phrase is accessible enough to be retrievable by form which is low in informativity (a pronoun) (Almor, 2000; Almor & Nair, 2007; Gundel, Hedberg, & Zacharski, 1993). Making wh‐phrases more specific boosts their accessibility, making retrieval by a pronoun more acceptable (Ariel, 2001; Gernsbacher, 1989).

## 5 Conclusion

Linguistic phenomena are characterized in the PA in terms of corresponding syntactic, semantic, and phonological representations, linked to nonlinguistic cognitive representations and processing mechanisms. The PA thus views language's internal components as interconnected in a similar way to how language is connected to the rest of the mind (Culicover, 2015; Jackendoff, 2002). While details about these connections remain to be explored, the PA provides a framework that is “minimalist” and allows for integration of linguistic theory with broader investigations about cognitive science and biology.

## Notes

1 Due to space limitations, we only discuss (i)–(iii). For (iv), see Culicover (2021).

2 The SiSx program is similar to HPSG (Pollard & Sag, 1994) and LFG (Bresnan, Asudeh, Toivonen, & Wechsler, 2016). For a more thorough comparison, see Varaschin (2023).

3 It is this expressiveness that makes the PA particularly well‐suited to accounts of language variation and change (Culicover, 2021).

4 Following Jackendoff (2002), we assume that the system of sem interfaces with a separate system that represents the modality‐independent understanding of the spatial structure of objects. This spatial structure allows us to talk about what we see and to distinguish the lexical meanings of words like *zebra* and *horse* without needing to appeal to ad hoc semantic primitives like $\mathrm{has}-{\mathrm{stripes}}^{\prime }$ (Jackendoff, 1987, 2012). Geometrical and topological aspects of human reasoning are explained in terms of spatial structure, while more discrete and logical aspects are explained in terms of sem. Some theories of semantics are incomplete precisely because they fail to recognize the independence of these two levels. We assume that defeasible pragmatic inferences like implicatures do not require an extra level of representation, but can be defined as mappings from sem into sem (Jackendoff, 1983, 105). This preserves the notion that words and constructions are conventionally/grammatically associated with particular semantic structures (i.e., their “literal meaning”).

5 Another open issue is whether lexical meanings are atomic or have an internal hierarchical structure that bottoms out in a finite repertoire of primitive concepts like ${\mathrm{person}}^{\prime }$, ${\mathrm{cause}}^{\prime }$, ¬, ∃, ∀, and so on (Jackendoff, 1983, 1990). For convenience, we represent word meanings as basically atomic and only sentence meanings as hierarchically structured.

6 The same basic reasoning applies to (i), which is also problematic for syntactic approaches (Lipták, 2020).

(i) A: Where is Amy running?
  B: To school, but she is not running (meaning “Amy is moving towards the school, but she is not running,” not “Amy is running to the school, but she is not running.”)

*To school* matches *where* in the antecedent. Crucially, however, the part of the remaining meaning of the antecedent that is identical to the semantics of the fragment is not “Amy is running to x,” given that B's reply contains a correction regarding Amy's manner of motion. The meaning is something more abstract, like “Amy is moving towards x.” Jackendoff (1983) argues that this is in fact the basic semantic structure of manner of motion verbs like *run*. Jackendoff (1990, 88) hypothesizes that different manners of motion are not represented at the level of sem, but only at the level of spatial structure, which, as (i) shows, is not relevant for the purposes of computing ellipsis. It is difficult to see how this effect can be captured in a system without parallel levels of representation for linguistic expressions.

7 Plausibly, a referent is more accessible to the extent that it is more likely to be mentioned in the context at hand (Arnold, 2010; Arnold & Tanenhaus, 2011; Culicover, Varaschin, & Winkler, 2022).

8 The RUH is also in line with neurolinguistic findings showing that the language system in the brain has a short temporal receptive window, being mostly sensitive to “local” stimuli (Blank & Fedorenko, 2020; Jacoby & Fedorenko, 2020).

9 Characterized as an instance of freezing by Wexler and Culicover (1980).
